# The importance of polymorphisms in the genes encoding glutathione S-transferase isoenzymes in development of selected cancers and cardiovascular diseases

**DOI:** 10.1007/s11033-023-08894-4

**Published:** 2023-10-11

**Authors:** Katarzyna Grussy, Magdalena Łaska, Wiktoria Moczurad, Magdalena Król-Kulikowska, Milena Ściskalska

**Affiliations:** 1 Student Society of Laboratory Diagnosticians, Faculty of Pharmacy, Wroclaw Medical University, Borowska 211a, 50-556 Wroclaw, Poland; 2 Department of Pharmaceutical Biochemistry, Faculty of Pharmacy, Wroclaw Medical University, Borowska 211a, 50-556 Wroclaw, Poland

**Keywords:** Glutathione S-transferase, Polymorphism, Colorectal cancer, Prostate cancer, Breast cancer, Cardiovascular disease

## Abstract

Glutathione S-transferases are a family of enzymes, whose main role is to detoxify cells from many exogenous factors, such as xenobiotics or carcinogens. It has also been proven that changes in the genes encoding these enzymes may affect the incidence of selected cancers and cardiovascular diseases. The aim of this study was to review the most important reports related to the role of glutathione S-transferases in the pathophysiology of two of the most common diseases in modern society – cancers and cardiovascular diseases. It was shown that polymorphisms in the genes encoding glutathione S-transferases are associated with the development of these diseases. However, depending on the ethnic group, the researchers obtained divergent results related to this field. In the case of the *GSTP1* A/G gene polymorphism was shown an increased incidence of breast cancer in Asian women, while this relationship in European and African women was not found. Similarly. In the case of cardiovascular diseases, the differences in the influence of *GSTM1*, *GSTT1*, *GSTP1* and *GSTA1* polymorphisms on their development or lack of it depending on the continent were shown. These examples show that the development of the above-mentioned diseases is not only influenced by genetic changes, but their pathophysiology is more complex. The mere presence of a specific genotype within a studied polymorphism may not predispose to cancer, but in combination with environmental factors, which often depend on the place of residence, it may elevate the chance of developing the selected disease.

## Background

In a constantly changing environment there is the exposure to many substances of external origin, including heavy metals, pesticides, organic solvents or even tobacco smoke that are not neutral to the human body. Exposure to these factors leads to the formation of free radicals—chemical compounds containing unpaired electrons that can easily react with chemical substances and cause tissue damage if they appear in excess [1]. However, the human body has developed certain mechanisms that allow it to neutralize free radicals. The components of these mechanisms are antioxidants, which can be divided into enzymatic and non-enzymatic [2]. The first group includes enzymes, such as superoxide dismutase (SOD), catalase (CAT), glutathione peroxidase (GPx) or glutathione S-transferase (GST). The second group includes polypeptides (thioredoxins, glutaredoxins, sulfiredoxins), minerals (zinc, copper, selenium, manganese) and low molecular weight antioxidants, among others, glutathione (GSH), metallothioneins (MTs) or ascorbic acid (vitamin C) [3–5].

In fact, there is a whole family of glutathione S-transferases (GSTs), enzymes whose main functions include cell detoxification and the removal of many physiological substances and xenobiotics [6]. GSTs catalyse the nucleophilic addition of γ-glutamylo-cysteinylo-glycine to electrophilic centres in organic compounds to form the glutathione conjugates that are more soluble in water and therefore can be eliminated more easily from the body. GSTs are present in both, eukaryotes and prokaryotes; isoenzymes of GST were found in the cytoplasm, microsomes and mitochondria [7, 8]. Due to the importance and the role of this enzyme in the organism, the changes in the genes coding for them can have an influence on the susceptibility to diseases such as asthma, atherosclerosis, allergy, cardiovascular diseases, neurodegenerative diseases and diabetes [9]. There is more and more the evidences supporting the role of GSTs and their polymorphisms in the development of certain cancers, as well as chemotherapeutic resistance [10–12]. The level of GSTs expression seems to be an important factor influencing on the sensitivity of cells to toxic substances [9].

Interestingly, although it has been proven that specific changes in the genes encoding GSTs may have a real impact on the development and the course of cancers, in the published studies the discrepancies depending on the studied population often are occurred. This is most likely due to the degree of the exposure to environmental factors among different ethnic groups [13, 14]. This review is a summary of the current state of knowledge on GSTs, their functions and the role of polymorphisms in the genes encoding GSTs in the development of selected diseases affecting modern humanity. This review is also intended to encourage the researchers to further studies focused on GSTs-dependent treatments, what could have a clinical significance.

## The structure of GSTs

GSTs are proteins with a wide variety of functions that can be found in all tissue of the organism. GSTs are present in most cells; they are common in the human kidneys, liver and intestines, with cytosolic GST having the highest activity in the liver. GST belongs to the class of phase II enzymes—EC 2.5.1.18. There are three superfamilies of this enzyme: soluble GST (cytosolic fraction), mitochondrial fraction and microsomal GST known as the membrane associated proteins involved in eiocosanoid and glutathione metabolism [15].

Soluble GSTs consist of two subunits (25 kDa), which are divided into two domains: the N-terminal α/β forming the G region and the α-helical forming most of the H region, which is responsible for the binding of the electrophilic substrate. The cytosolic GST (soluble) is main isoenzyme of GSTs family. Many soluble GSTs are characterized by a crystalline structure [16]. Among them, eight classes are distinguished: alpha (α), mu (µ), sigma (σ), omega (ω), pi (π), theta (θ), zeta (ζ) and kappa (κ). The main classes are α, µ and π. Isoenzymes in the above classes are characterised by the immunological reactivity, the primary structure (sequence) and the protein substrates specificity. GST-µ (GSTM), GST-α (GSTA), GST-π (GSTP) and GST-θ (GSTT) are the most abundant, similarly as their the allele variability in human population. This diversity influences on the interindividual differences in the metabolism of xenobiotics and drugs [17].

The GST-α class is encoded by 5 genes (*GSTA1*, *GSTA2*, *GSTA3*, *GSTA4*, *GSTA5*) and 2 pseudogenes (*GSTA6P* and *GSTA7P*). These genes are located on the short arm of chromosome 6 (6p12) and they are the most expressed in the liver. The genes encoding GST-µ, located on the short arm of chromosome 1 (1p13.3), consist of: *GSTM1*, *GSTM2*, *GSTM3*, *GSTM4* and *GSTM5*. *GSTM2* was found in the muscles and *GSTM3* – in the brain. The GST-π class is encoding by *GSTP1* in humans, which is located on the long arm of chromosome 11 (11q13), while the genes encoding GST-θ are located on the long arm of chromosome 22 (22q11.2). Among these one, the following genes are distinguished: *GSTT1*, *GSTT2* and *GSTT2B*. The sigma class is encoded by gene located on the long arm of chromosome 4 (4q21-22) [15, 18]. In mammalian, GST α, µ and π classes are characterized by high affinity for matrices, e.g. S-glutathione-agarose, and the other classes show the opposite relationship (weak binding or the lack of it) [16]. These differences allow using the GST-GSH binding affinity in protein recombination systems as GSTs, GSTs-α, GSTs-µ and GSTs-π fusions. They are also highly expressed and easily purified, what has resulted in the frequent use of these human classes of glutathione-S-transferase in studies [16].

The GST-θ differs significantly from the GSTs of the α, µ, and π classes. The discrepancies are visible in the sequence, structure and catalytic activity of the isoenzymes [16]. GST-θ is evolutionarily different from the classes above mentioned, as evidenced by the fact that GST-θ, in contrast to other classes, is present in plants. It is likely that GST-θ was distinguished from the ancestors of GSTs-α, GSTs-µ, GSTs-π long before the isolation of this isoenzyme from animals and plants [16]. In the human genome, mainly *GSTT1-1*, *GSTT2-2* and possibly *GSTT2B-2B* are expressed [16].

**Scheme 1:**
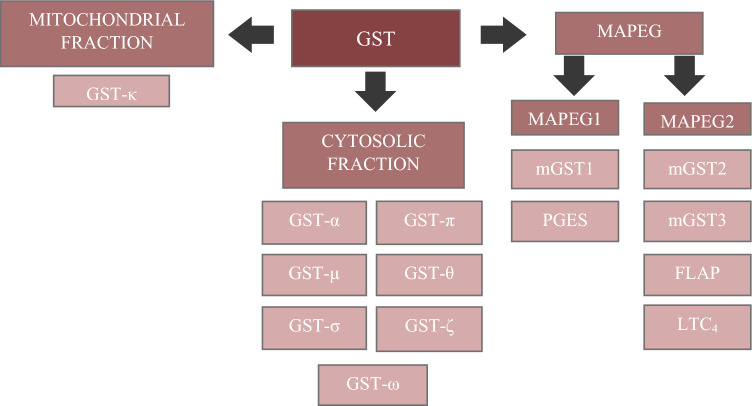
GST family and cellular localization of its isoenzymes. GST—S-glutathione-transferase; GST-α—alpha class glutathione-S-transferase; GST-π—class pi glutathione-S-transferase; GST-µ—mu class glutathione-S-transferase; GST-θ—theta class glutathione-S-transferase; GST-σ—sigma class glutathione-S-transferase; GST-ζ—zeta class glutathione-S-transferase; GST-ω—omega class glutathione-S-transferase; GST-κ—kappa class glutathione-S-transferase; MAPEG—membrane protein; MAPEG1—membrane protein, subfamily 1; MAPEG2—membrane protein, subfamily 2; mGST1—mitochondrial glutathione-S-transferase 1; PGES—5-lipoxygenase activating protein; mGST2—mitochondrial glutathione-S-transferase 2; mGST3—mitochondrial glutathione-S-transferase 3; FLAP—prostaglandin E synthase; LTC4—C4 leukotriene synthase [17, 21, 22]

Another superfamily of GSTs constitutes the membrane-associated proteins involved in eicosanoid and glutathione metabolism (MAPEG). MAPEG are proteins that play an important role in the metabolism of arachidic acid, occurring in both, eukaryotes and prokaryotes (excluding archaebacteria) [19]. Two families can be distinguished in this superfamily: MAPEG1 and MAPEG2. The MAPEG1 family includes mGST1 and PGES (prostaglandin E synthase), while the MAPEG2 family – mGST2, mGST3, FLAP and LTC4 synthase [20]. Thanks to electron crystallography, it was found that proteins from the “MAPEG” superfamily are composed of four transmembrane helices that are grouped into trimers (21). The isoenzymes belonging to GST family in the diagram Scheme 1 were shown.

## Functions of GSTs

Glutathione-S-transferase is an enzyme involved in the xenobiotic metabolism; it catalyses the biotransformation of substances containing various functional groups (both endo- and exogenous compounds), including quinones, epoxidones and hydroperoxides [2]. GSTs catalyse the detoxification of endogenous hydroperoxides, e.g. hydrogen peroxide or lipid hydroxide, and exogenous compounds such as cumene hydroperoxide. Catalytically active GSTs carry out thiol transferase reactions. GST isoenzymes catalyse the process of glutathione-dependent isomerization, which is required for the synthesis of some prostaglandins, steroid hormones or tyrosine catabolism [16].

However, the predominant role of GST in metabolism is based on catalysing the reaction of reactive electrophiles with glutathione, thus reducing their toxic effect on cellular components, especially on the proteins and nucleic acids (GST protects e.g. chromosomes from damage by oxidative stress). Through this mechanism, GST is involved in the pathways that inactivate and eliminate many drugs being the electrophilic compounds (or their precursors), including chemotherapeutic agents used in anti-cancer therapy [16]. Another mechanism of GST action is based on the using of electrophiles as substrates for the conjugation with glutathione. Electrophiles are derived from the metabolism of drugs, e.g. felbamate, the phenylpropenal metabolite (used in the treatment of epilepsy). Glutathione adducts efflux outside the cell by transporters, e.g. MRP1 (multidrug resistance-associated protein 1), and then excreted in the urine or bile in the form of mercapturic acids (whose formation precedes the separation of the glutamine and glycine residues from the adduct and the acetylation of free amino group in cysteine). Occasionally, the conjugation reactions catalysed by GST can lead to the formation of reactive intermediates [16].

GSTs also play a role in the cellular response to oxidative stress. They are important in the processes of oncogenesis, cancer progression, drug resistance, as well as the metabolism and biosynthesis of prostaglandins, leukotrienes and steroids [17]. It has been proven that GST isoenzymes affect cell signalling pathways responsible for cell proliferation and death. GSTs are crucial determinants of the pro-inflammatory conditions in the course of cancer. It was demonstrated in animal model using the GSTP -/- knockout mice and their wild type counterparts—the GSTP -/- knockout mice showed a faster development of TPA (13-acetate-12-O-tetradecanoylphorbol) induced by human papilloma compared to their wild type [17].

GST-α, GST-µ, GST-π and GST-θ are involved in the detoxification of xenobiotics, drugs and carcinogens [21]. Moreover, many polymorphisms related to the GSTs genome cause changes in the molecular structure of the enzyme, affect its stability and the variability of enzymatic activity and detoxification capacity, which may have a detrimental effect on cells and lead to various types of cancer. GST-α protects the cells against the harmful effects of peroxidation products and reactive oxygen species (ROS). This class includes the enzymes with glutathione peroxidase activity, responsible for the detoxification of lipid peroxidation products. In addition, GSTs-α is recognized as an indicator of liver damage. Its decreased activity occurs in acute liver damage [15]. The µ class influences on the drug efficacy and can modify the toxicity of xenobiotics [15]. Functional GST-µ can prevent or repair damage to DNA by inactivating carcinogens and lipid peroxidation products. Deletion of the *GSTM1* gene can lead to inactivation of GST-µ, altering a resistance to poisons and carcinogens, what can result in the loss of detoxification capacity and increasing the risk of cancer development [23]. The θ class may also play a role in human carcinogenesis. Its main roles are related to detoxification or antioxidant processes, therefore the change of structure of GST-θ molecule as an effect of SNP (single nucleotide polymorphism) is strongly associated with chronic diseases. The *GSTT1* and *GSTM1* gene polymorphisms consist of a deletion (null) and a functional (positive) genotype, which means that the null genotype weakens the response to xenobiotics [15, 23]. For example, the functional *GSTT1* genotype encodes GST-θ isoenzymes and is characterized by a high detoxification capacity against the halogenated hydrocarbons and pesticides. Deletion in the *GSTT1* gene will result in a deficiency (lack) of GST-θ activity, thus affects the detoxification capacity of the individual and increases its susceptibility to carcinogenic compounds [23]. A relationship between deletion in the *GSTT1* and *GSTM1* genes and an increased risk of many types of cancer occurrence (liver cancer, breast cancer, cervical cancer, head and neck cancer, oesophageal cancer, oral cancer, lung cancer) has been shown [23]. The π class is involved in the protection of cells against carcinogens and cytotoxics. It was reported that GST-π inactivation was often observed in human cancers (liver cancer, breast cancer, prostate cancer, leukaemia). Epigenetic modifications in the *GSTP1* gene can be recognised as biomarkers for the diagnosis of cancers in its early stages, and thus for prophylaxis or treatment monitoring [15].

## The influence of GST polymorphisms on the increased risk of selected cancers occurrence

Among the known isoenzymes of GST, the most studied polymorphisms in relation to cancers are the SNP in the *GSTM1*, *GSTT1* and *GSTP1* genes [24]. Three polymorphisms were identified within the *GSTM1* gene, which is located on chromosome 1p13.3. One of them is the null mutation resulting in the lack of a protein product, what is associated with a complete lack of enzymatic activity [25]. In contrast, the GSTM*A and GSTM*B polymorphisms are associated with the C519G substitution (asparagine is substituted with lysine at position 173.7), while the lack of scientific evidence for a functional difference between these polymorphisms means that they are classified together as phenotype 7. The *GSTT1* gene is localized on chromosome 22q11.2 and includes two alleles: GSTT*1 active allele and GSTT*0 null genotype. It was shown that the subjects with a homozygous deletion of the *GSTT1* locus (*GSTT1* 0/0) are characterised by the lack of the activity of the appropriate enzyme [26]. In turn, the polymorphism in the *GSTP1* gene, located on chromosome 11q13, is most often a single nucleotide mutation within exon 5. As a result of this mutation, *GSTP1* Ile/Ile, Ile/Val and Val/Val genotypes may arise [25], what results in the formation of protein characterised by reduced enzymatic activity [24].

### Influence of GST polymorphisms on the risk of colorectal cancer occurrence

According to data from 2021 reported by the American Cancer Society, colorectal cancer (CRC) is one of the most common types of cancer in both women and men (approx. 8% of cases) [27]. The most common causes of CRC are spontaneous mutations accompanied by environmental factors. The cases of hereditary occurrence of CRC constitute only about 5% [28]. Based on previous studies, it was found that the polymorphisms in the genes encoding GSTs are characterized by a small, but significant risk of developing CRC [29]. SNPs in these genes often lead to a change or even complete lack of enzyme activity of GSTs, which, due to their important role in the detoxification of heterocyclic aromatic amines and polycyclic aromatic hydrocarbons, may affect the carcinogenesis pathways and result in CRC [30]. It was observed significant differences between the prevalence of polymorphisms in the genes encoding GSTs and cancer risk among different populations. Based on studies conducted on the Central European Caucasian population was noted the relation between the occurrence of the heterozygous Ile105Val genotype (SNP rs1695 in the *GSTP1* gene) and a reduced risk of CRC. On the other hand, the studies focused on the polymorphisms in the *GSTM1* and *GSTT1* genes were shown no statistically significant relationship with the risk of CRC occurrence [31].

The study conducted on the Polish population have shown that the *GSTT1* gene polymorphism was correlated with a higher degree of malignancy in patients with CRC. Additionally, it was shown that the *GSTM1* null genotype was associated with more frequent metastasis to the lymph nodes [32]. In turn, in another study conducted on this population were not proved that polymorphisms in the genes encoding GST directly predisposed to the development of cancer. However, it was found that the presence of polymorphisms in the genes encoding this enzyme can contribute to an increased risk of CRC development in the case of interaction with other factors, such as diet or smoking [33].

In India, studies have confirmed the association of *GSTM1*-null genotype with an increased risk of rectal cancer and *GSTT1*-null genotype with an increased risk of colon cancer. It was also found that the simultaneous presence of polymorphisms in *GSTM*, *GSTT1* and *GTTP1* genes may contribute to the development of CRC [34]. The influence of the SNP rs1695 in the *GSTP1* gene was observed in the Tunisian population. It was shown that the individuals with the 105Val allele have an increased risk of developing CRC than those with the 105Ile allele. In this study was not confirmed an increased susceptibility to CRC in the presence of *GSTT1* and *GSTM1* gene polymorphisms [35]. On the other hand, in the case of the Chinese population, it was observed that all above-mentioned polymorphisms were associated with the elevated risk of CRC occurrence in smokers due to carcinogens present in cigarette smoke [36]. In another study conducted on the Chinese population, it was found that *GSTT1* and *GSTM1* gene polymorphisms may increase the risk of CRC, while *GSTP1* gene polymorphism was not associated with an increased risk of disease development [37].

In conclusion, the *GSTM1* null genotype is associated with an increased risk of CRC in Asians and Caucasians, according to a comprehensive meta-analysis [38]. Moreover, previous meta-analyses also indicated an association between the Asian population (particularly Chinese) and an increased probability of CRC occurrence. For example, Huang et al. [39], based on the analysis of 55 studies, have shown that the *GSTM1* null genotype is a pathological factor in the development of CRC. Zhong et al. [40] were found that the *GSTT1* null genotype may contribute to the development of CRC. Similar conclusions were also drawn by other researchers [41], however, in a meta-analysis by Wang et al. [42] no associations between the *GSTM1* null and *GSTT1* null genotypes and an increased risk of CRC were found. Although most meta-analyses indicate that the *GSTT1* null genotype and the *GSTM1*/*GSTT1* null genotype may predispose to an increased risk of cancer development in East Asian population [38], this hypothesis should be taken into the account with some caution. The differences in results of the research focused on the polymorphisms in the genes encoding GST may result from ethnic diversity and the insufficient power of individual analyses, too. The studying of the ethnic influence on the development of a given disease requires the use of a sufficiently large cohort.

### The influence of GST polymorphisms on the of prostate cancer occurrence

According to the latest data, prostate cancer is the most common cancer in men, accounting about 26% of all cancers and 11% of cases of death [27]. Malignant neoplastic transformation takes place in several stages: in the beginning, there is intraepithelial neoplasia, from which a local prostate cancer is developed, and then, advanced prostate adenocarcinoma. The final stage of neoplasm is metastatic prostate cancer, which is the main cause of death due to this cancer [43]. The possible risk factors for the development of prostate cancer may include older age, androgen stimulation and ethnicity. It was shown that polymorphisms in the genes encoding GSTs may also predispose to the development of prostate cancer due to insufficient detoxification of environmental carcinogens [44]. The meta-analysis conducted in 2012 was found that *GSTM1* null genotype, *GSTM1* and *GSTT1* double null genotype or *GSTT1* null genotype and rs1695 polymorphism within the *GSTP1* gene can correlate with an increased risk of cancer development [45]. Other studies have shown that men with at least one version of the SNP in the *GSTP1* gene: rs1138272 (*Ala/Val + Val/Val) or rs1695 (*Ile/Val + Val/Val) have an elevated risk of prostate cancer [46]. The effect of genetic polymorphisms on the development of this cancer, as in the case of CRC, is depended on the population. In meta-analysis, it was concluded that the *GSTM1*-null genotype was significantly associated with the risk of cancer development for the Asian population, but no significance effect of this SNP in the Caucasian population was noted [25]. In turn, in the case of studies conducted on the Algerian population, it was observed that the *GSTM1*-null genotype may increase the susceptibility to this cancer, but the *GSTT1* null genotype has not contributed to the development of prostate cancer [44]. Similar results were obtained for the Asian population, in which it was shown that the *GSTM1* null genotype was associated with an increased risk of prostate cancer occurrence in China and Korea. In turn, for the *GSTT1* null genotype, a similar relationship was not demonstrated [47]. The studies conducted on the Polish population has shown that polymorphisms in the genes encoding GSTs were not related to the risk of prostate cancer development [48]. *GSTM1* gene polymorphism was associated with the increased risk of this cancer occurrence for various ethnic groups, while in the case of *GSTT1* gene polymorphism, the correlation related to the risk of cancer development in the Caucasian, Asian or African-American populations was not observed [49].

### The influence of GST polymorphisms on the risk of breast cancer occurrence

Breast cancer is the most common cancer in women; based on statistical analyses carried out in the United States, 30% of new cases of it were recorded in 2021. Breast cancer is the cause of death in 15% of cases [27]. Breast cancer incidence rates continue to rise by around 0.5% per year, what can be partly attributed to the continued decline in fertility rates and increased body weight [50]. There are many causes that may increase probability of breast cancer development, such as DNA damage and mutations. Moreover, the risk of breast cancer increases with age [51]. The SNPs in the genes encoding GSTs that are studied to stratify the risk of development of this cancer, most often are focused on *GSTP1* gene, of which rs1695 and rs1138272 are the most common variants of this gene. It has been established that there is a significant relationship between the genetic variability of rs1695 and the risk of breast cancer in homozygotes [52]. Detection of this polymorphism may help in screening the high-risk population and steer the individual therapy [53]. Based on a meta-analysis conducted in 2013 was found the correlation between the *GSTP1* A/G gene polymorphism and the susceptibility to breast cancer in the Asian population. However, this polymorphism was not associated with the risk of breast cancer in the European or African population [54]. In another meta-analysis, it was also shown the association between the *GSTM1* and *GSTP1* gene polymorphisms and an increased risk of breast cancer occurrence in the Asian population (especially in East Asia). However, a similar relationship was not found for the *GSTT1* gene polymorphism [55]. In other study have also shown that the rs1695 polymorphism in the *GSTP1* gene, the *GSTM1* null genotype and the *GSTT1* null genotype were also associated with an increased incidence of breast cancer [56]. In the study carried out on the Mexican population was found a relationship between the occurrence of the *GSTM1* null genotype and the risk of breast cancer [57]. Another analysis conducted on the Filipino population have found that *GSTM1* and *GSTT1* null genotypes can be to recognise as a risk factors for development of breast cancer, but this risk may increase when these genotypes are combined with lifestyle or environmental factors [58]. Based on the available literature, it is impossible to clearly confirm or exclude the role of SNPs in the genes encoding GST in the development of breast cancer. Although some studies indicate that ethnic and environmental factors, in addition to genetic factors, can also influence on disease progression, the caution should be exercised in drawing too hasty conclusions. Despite the fact that the results of meta-analyses were reported in this review, it should be remembered that some of them are relatively new studies that require further confirmation.

### The influence of GST polymorphisms on the risk of chronic myeloid leukaemia occurrence

Chronic myeloid leukaemia (CML) is a myeloproliferative cancer characterized by clonal growth of a multi-potential myeloid stem cell in the bone marrow. The CML development has been associated with the cytogenetic change t(9,22)(q34, q11) resulting in the formation of the *BCR-ABL1* gene fusion [59, 60]. However, the occurrence of CML is also an effect of the imbalance between exposure to carcinogens and the ability of enzymatic systems to detoxify of them [61]. The polymorphisms in the genes encoding GSTs may influence on the risk of CML development, but literature data about a specific genotypes that may predispose to cancer occurrence are contradictory. It was observed that the appearance of the *GSTT1*-null genotype enhanced near threefold the risk of CML in comparison with the subjects with both alleles in this gene [61]. The study conducted on the Syrian population has found a correlation between *GSTM1* null genotype (alone or in combination with *GSTT1* null genotype) and the risk of CML occurrence [62]. Other researchers have also noted the association of *GSTT1* and *GSTM1* polymorphisms with the susceptibility to the development of CML [63]. However, in other study was shown that *GSTM1* and *GSTT1* were not associated with the risk of CML, while the variant of the rs1695 polymorphism in the *GSTP1* gene may enhance the risk of this disease [64]. It was observed that the interaction of polymorphisms in the genes encoding GSTs with cigarette smoking plays an important role in the development of CML [65].

Moreover, in meta-analysis was shown that genetic variants in the genes encoding GSTs may not only play a role in the development of CML, but also affect the response of patients to tyrosine kinase inhibitor (TKI) treatment. The occurrence of the GG genotype (Ile105Val in the *GSTP1* gene) was associated with a worse response to treatment compared to wild-type homozygotes [66]. In another study was shown that this genotype and the presence of the *GSTM1* gene polymorphism are also associated with a worse response of patients to treatment. However, these results were on the verge of statistical significance [67].

### The summary of the influence of GST polymorphisms on the risk of cancer occurrence

In conclusion, there are the findings that polymorphisms in the genes encoding GSTs may predispose to the cancers’ occurrence, but significant differences are observed in individual populations. These differences may result from the exposure of a given ethnic group to harmful environmental factors or be caused by insufficient power of analyses. Despite the fact that there have already been many meta-analyses indicating the role of the polymorphisms in the genes encoding GST isoenzymes in the cancer development, the caution should be exercised before drawing unambiguous conclusions. The reviewed studies are relatively new reports, therefore the hypotheses presented in them should be treated as working ones. The confirmation of the obtained results should be carried out by multiple hypotheses testing. Moreover, a noticeable influence of the environmental or ethnic factors can also be caused by underpowered analysis. To confirm this effect, a sufficiently large cohort should be included in the study and the analysis should include rare coding mutations.

## The influence of GST polymorphisms on the risk of cardiovascular diseases occurrence

Cardiovascular diseases are one of the major causes of death in the modern world. Both, environmental and genetic factors play a role in their pathogenesis [68, 69]. The risk factors for the development of cardiovascular diseases include: hypertension, disorders in the lipid metabolism (such as hypercholesterolemia, hypertriglyceridemia and low concentration of HDL (high-density lipoprotein)), diabetes, obesity, low physical activity, alcohol consumption, smoking, menopause and exposure to xenobiotics [70–72].

Numerous studies have proved that polymorphisms in the genes encoding GST isoenzymes are recognised as the risk factors for development of these diseases [73–76]. Disrupting the antioxidant function of GST, as a result of the changes in the genes encoding GSTs, can lead to an increase in the amount of free radicals in the body. The increasing oxidative stress has a destructive effect on lipids, proteins and nucleic acids, causing a tissue damage [68, 77]. The influence of free radicals on the human body is particularly noticeable in the pathogenesis of hypertension, because ROS directly impair the proper functioning of vascular endothelium. Under physiological conditions, the endothelial cells produce NO, which is formed from L-arginine by the endothelial nitric oxide synthase (eNOS). NO, as a result of its action, stimulates soluble guanylonic cyclase (sGC) increasing the concentration of cGMP in the body. This process results in a transient decrease in the level of calcium ions in the cell and the relaxation of smooth muscles of blood vessels [78]. ROS, by binding to NO, can contribute to the formation of the highly reactive radical – peroxynitrite (ONOO^−^), which impairs the proper function of eNOS [78]. The harmful effect of ONOO^−^ is also based on the oxidation of the eNOS cofactor to BH_2_ (dihydrobiopterin), which leads to the decoupling of eNOS. These processes lead to prolonged vasospasm and development of hypertension.

The polymorphic changes in the genes encoding GST isoenzymes may cause metabolic effects, which are recognised as a risk factors for hypertension occurrence. Studies conducted on the North Indian population have shown that *GSTT1* null genotype and *GSTM1* positive genotype are associated with the development of essential hypertension in the subject with impaired lipid profile [75]. In other studies were shown that *GSTM1*-null/*GSTT1*-null genotype may be also associated with the disturbing of lipid metabolism, what was reflected in higher body weight, waist circumference, total cholesterol, LDL and HDL compared to the individuals with the *GSTM1*-active/*GSTT1*-active genotype. A greater waist circumference were found in the subjects with the *GSTT1*-null genotype compared to those with the *GSTT1-*positive genotype. Noteworthy is the fact that the subjects with hypertension and the *GSTM1*-null genotype had lower triglyceride concentration than the subjects with the *GSTM1*-positive genotype [76]. On the other hand, the study conducted on the United Arab Emirates population did not show an increased risk of hypertension among the individuals with the *GSTT1*-null/*GSTM1*-null genotype [73].

There are meta-analyses demonstrating that *GSTM1* null, *GSTP1* null and *GSTT1* null polymorphisms were significantly associated with an increased risk of coronary artery disease (CAD) in overall population [79]. Null polymorphisms of *GSTM1*, *GSTP1* and *GSTT1* genes can result in a diminished gene expression level and a reduced enzymatic activity of GST. Consequently, it can have a biological effect, what affect the risk of CAD [79]. Additionally, meta-analysis conducted by Su et al. [79] has found that *GSTT1* null polymorphism was significantly associated with the risk of CAD in Caucasians and East Asians. In other meta-analysis was found that *GSTM1* null genotype and *GSTP1* (Ile105Val) polymorphism was significantly associated with CAD risk, similarly to dual null genotype of *GSTM1-GSTT1* genes [80]. However, Sobha et al. [80] have shown no significant association between the *GSTT1* null genotype with CAD, but the potential interaction between *GSTT1* null genotype and smoking in meta-analysis conducted by Song et al. [81] was found. Liu et al. [82] have shown no association between the *GSTM1* null polymorphism and CAD, but increased risk of CAD in the smoking population with the GSTM1 null genotype was observed. Their findings confirm the results of the meta-analysis (involving more than 47,500 subjects) in which overall lack of association between *GSTM1* genotypes and coronary heart diseases was shown. On the other hand, in this study was found that *GSTM1* null genotype when combining with smoking history may contribute to disease susceptibility [83].

The role of polymorphism in the genes encoding GSTs as a risk factor for ischemic heart disease was also examined. It was found that *GSTM1*, *GSTP1* and *GSTT1* null genotypes are associated with the increased risk of its occurrence [79]. However, among the Indian population of South Africa, a correlation between the *GSTM1*-null genotype and the A105 allele of *GSTP1* and ischemic heart disease was found [84]. On the other hand, studies on the Taiwanese population did not reveal that the *GSTM1*, *GSTT1*, *GSTP1* and *GSTA1* polymorphisms were associated with ischemic heart disease [85].

*GSTP1* genetic polymorphism (A, B, C alleles) is recognised as a risk factor for myocardial infarction during surgery on this organ. It has been found that the presence of the B allele may prevent from myocardial infarction, while the increased risk of myocardial infarction is associated with the presence of the C allele [86]. It was also shown that the GSTP1*Val variant plays a significant role in the occurrence of another disease—heart failure—especially when the GSTA1*B allele is additionally occurred [76]. The table below shows the results of studies focused on the polymorphisms in the genes encoding GST isoenzymes as related to the occurrence of cardiovascular diseases (Table 1).

**Table 1:** Relationship of the polymorphisms in the genes encoding the GST isoenzymes with the occurrence of cardiovascular diseases

Population	Genetic variants in the genes encoding the GST isoenzymes	Odds ratio	Year of publication	References
Hypertension				
India	*GSTM1*-null	1.50 (1.029–2.194)	2011	[100]
	*GSTT1*-null (smokers)	2.89 (1.17–7.17)		
Italy	*GSTT1*-null (women)	3.25 (1.78–5.95)	2011	[101]
Korea	*GSTM1*-null	Lack of information	2011	[102]
Korea	*GSTM1*-positive/*GSTT1*-positive	1.719 (1.076–2.745)	2012	[103]
	*GSTM1*-null/*GSTT1*-positive	1.637 (1.087–2.466)		
Slovenia	*GSTM1*-null	1.7 (1.2–2.4)	2014	[104]
	*GSTT1*-null	1.5 (1.1–2.0)		
India	*GSTT1*-null	3.67 (1.19–11.31)	2015	[75]
	*GSTM1*-positive	2.15 (1.29–3.56)		
West Africa, Burkina Faso	*GSTT1*-null	1.79 (1.25–2.58)	2020	[76]
	*GSTM1*-active/*GSTT1*-null	2.33 (1.50–3.65)		
Ischemic heart disease				
South African Indians	*GSTM1*-null	2.386 (1.137–5.009)	2012	[84]
Mixed	*GSTM1*-null	1.37 (1.11–1.70)	2020	[79]
	*GSTT1*-null	1.23 (1.03–1.46)		
	*GSTP1*-null	1.23 (1.02–1.48)		
Heart failure				
Mixed	GSTP1*Val	1.7 (1.0–2.9)	2019	[74]
	Combined *GSTA1*/*GSTP1* (rs1695) *AB + BB/*IleVal + *ValVal	2.2 (1.1–4.4)		
	*GSTM1*-null (smokers)	4.4 (2.2–8.9)		

It should be added that polymorphic variants in the genes encoding GST isoenzymes may underlie the pathophysiology of metabolic impairment that are the risk factors for cardiovascular diseases. In the study conducted on the Polish patients, it was found that polymorphisms in the genes encoding GSTs may be a risk factor influencing on the occurrence of type 2 diabetes (T2DM) at a young age, too. The authors have reported that it was influenced by the occurrence of the *GSTP1* Val/Val, *GSTT1*-null and *GSTM1*-null genotypes [87]. These findings were confirmed by meta-analysis, in which was noted that *GSTM1* null genotype was linked to a particularly increased risk of T2DM in Caucasians and the *GSTP1* IIe105Val polymorphism was related to a substantially increased T2DM risk in Indians [88]. Moreover, the *GSTM1* and *GSTT1* double null genotype was associated with substantially increased T2DM risk in Caucasians and Indians; the combined effects of the polymorphisms in the *GSTM1* and *GSTP1* genes were associated with higher T2DM risk in Caucasians [88].

The findings about the associations between polymorphisms in the genes encoding GST isoenzymes and the risk of cardiovascular diseases are inconsistent. Although GST isoenzymes play important functions, it was not confirmed that deficiency of one type of the GST isoenzymes is problematic, probably because of redundant antioxidative systems. Thus, not every change in the genes encoding GST isoenzymes causes the change to be phenotypically expressed. The genetic effect on cardiovascular diseases occurrence is coupled to changes in many genes, often with favourable environmental and ethnic conditions. The association of the genetic variants in the genes encoding GSTs, especially *GSTM1* cluster, with cardiac problem, such as coronary artery disease, ischemic heart disease or heart failure were described in several GWAS across different populations worldwide [89–92]. GWAS studies have shown a significant association between cardiovascular diseases and *LPL*, *FADS2* and *C6orf184* gene expression, what play a role for regulation of lipid levels [93]. Several large scale GWAS and meta-analyses for *GSTM1* gene have identified the association between this gene and different lipid traits and many candidate gene association studies, especially LDL-C levels, ApoB, HDL, ApoA1, triglycerides, high total cholesterol level and Lp(a) [89, 94–98]. Over the last decade, many GWAS have also reported that the variants at *GSTP1* gene cluster were associated with plasma HDL, LDL, ApoA1 concentration and diastolic blood pressure [94, 95, 97]. However, GWAS and meta-analyses have not shown the association between *GSTT1* gene and cardiovascular diseases. On the other hand, in the study of gene-environment interactions, it was shown that SNP in the *GSTO1* gene, which was related to arsenic metabolism, exhibited significant interactions with cardiovascular disease, coronary heart disease or stroke [99]. These findings indicate that there might be a considerable number of unrecognized processes and mechanisms involved in the genes encoding the GST isoenzymes, which when disrupted, could contribute to cardiovascular diseases.

**Scheme 2:**
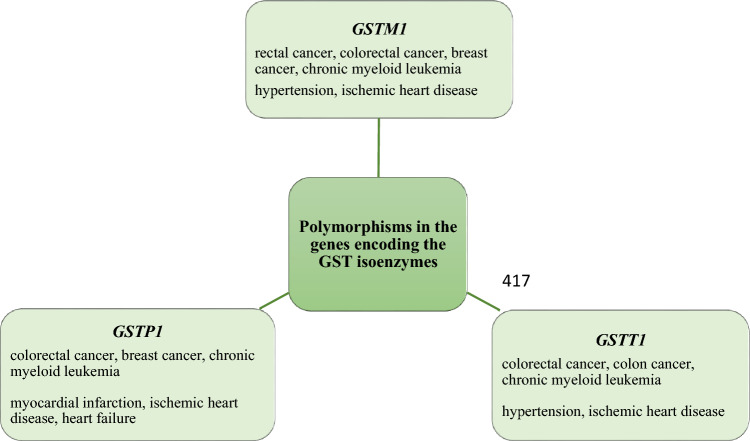
The relationship of polymorphisms in the genes encoding the GST isoenzymes with the selected diseases

The role of individual isoenzymes of GST in the development of selected diseases in Scheme 2 was summarized.

## Conclusions

It is well known that the GST isoenzymes belong to the most important detoxification systems among all living organisms. The disorders of expression of any genes may lead to the lack of the enzyme molecule or its partial inactivation. In some cases, genetic changes combined with factors such as diet, environmental factors, for example exposure to tobacco smoke, may modify the susceptibility to particular diseases, such as CRC, prostate cancer, breast cancer, CML and cardiovascular diseases. The polymorphisms in the genes encoding GSTs can contribute to above-mentioned diseases through induction of metabolic changes. The homozygous deletion of the *GSTT1* locus (null genotype) and the *GSTM1*-null genotype can result in a deficiency of the detoxification capacity of these isoenzymes and an increase in the susceptibility to carcinogenic compounds. It results in the occurrence of colorectal cancer, breast cancer and CML. Additionally, the occurrence of the *GSTM1*-null genotype can elevate the risk of prostate cancer. Similar molecular process of cancer development was shown in the case of SNP *rs1695* (*Ile/Val + Val/Val) and SNP rs1138272 (*Ala/Val + Val/Val) in the *GSTP1* gene that are associated with the lack of GST-π activity, and it can result in elevated the risk of prostate cancer development. SNP rs1695 in the *GSTP1* gene was also associated with an increased incidence of breast cancer (especially in Asian population) and development of CML. It was shown that the *GSTP1* Val/Val genotype, *GSTT1*-null and *GSTM1*-null genotypes can contribute to metabolic impairment. Above-mentioned polymorphisms can influence on plasma lipids levels, such as HDL, LDL, ApoA1 concentration, triglycerides, total cholesterol and Lp(a), which changes in concentrations are recognized as risk factors of cardiovascular diseases. It contributes to the occurrence of coronary heart disease, heart failure and myocardial infraction. As has been seen, polymorphisms in the genes encoding the GST isoenzymes are associated with two categories of the most common diseases—cancers and cardiovascular diseases, therefore further research into the molecular processes of these enzymes and their role in cells seems to be most warranted.

## Data Availability

Not applicable.
